# Validation of the revised Amyotrophic Lateral Sclerosis Functional Rating Scale in Poland and its reliability in conditions of the medical experiment

**DOI:** 10.1007/s10072-020-04565-5

**Published:** 2020-07-16

**Authors:** Stanisław Maksymowicz, Paula Kukołowicz, Tomasz Siwek, Agnieszka Rakowska

**Affiliations:** 1 Department of Psychology and Sociology of Health and Public Health, School of Public Health, Collegium Medicum of the University of Warmia and Mazury, Olsztyn, Poland; 2 Instytut Terapii Komórkowych S.A., Olsztyn, Poland; 3 Polish Economic Institute, Warsaw, Poland; 4 Department of Neurology, School of Medicine, Collegium Medicum of the University of Warmia and Mazury, Olsztyn, Poland; 5 University Clinical Hospital, Olsztyn, Poland

**Keywords:** Amyotrophic lateral sclerosis, ALSFRS-R, Validation, Medical experiment

## Abstract

**Introduction:**

Amyotrophic Lateral Sclerosis Functional Rating Scale-Revised (ALSFRS-R) is a basic tool for monitoring disease progression in amyotrophic lateral sclerosis (ALS). This study analyses the reliability of the Polish version of the ALSFRS-R as a tool to assess the health condition of patients with ALS and presents experience related to the use of this tool in monitoring the effects of experimental medical therapy.

**Materials and methods:**

The scale questionnaire was translated using the cross-translation method. The final tool was used by researcher, who was conducting the interview directly by telephone with patients and their caregivers and additionally compared with neurologopedic measurement. The health status of 60 patients was assessed between 4 and 7 times, which gives a total of 327 observations. Mean patient’s age was 57.5 ± 8.6. The division by sex was 23/35 (female/male). Patients’ health status and severity of symptoms varied. Statistical analysis was performed using explanatory factor analysis and Cronbach’s alpha.

**Result:**

Validation of the Polish version of the ALSFRS-R supports the reliability and internal consistency of scale. The scale proved also to be a proper tool for monitoring the course of the experimental medical therapy for patients with ALS. However, a qualitative evaluation revealed certain weaknesses of the scale, resulting from a different understanding of the functional assessment by the patient and by the medical specialist and cultural differences.

**Discussion:**

Although ALSFRS-R is a reliable enough for monitoring patient health, it seems reasonable to pay attention to some difficult points of the questionnaire and its improvement.

**Electronic supplementary material:**

The online version of this article (10.1007/s10072-020-04565-5) contains supplementary material, which is available to authorized users.

## Introduction

Amyotrophic lateral sclerosis (ALS) is a rare neurodegenerative disease damaging the central and peripheral motor neurons. The linear decline progress of the disease leads to paralysis, degeneration of speech, swallowing and breathing functions [1–3]. However, the feeling and cognitive functions of the nervous systems are preserved. Survival of ALS patients from first symptoms is about 3–5 years [4].

The clinical forms of ALS include the bulbar and the limb/spinal form [5]. In the bulbar form, already in the early phase of the disease, the muscles supplied by cranial nerves leading from the brain medulla (responsible for swallowing and for tongue and soft palate functions) are affected. In the limb/spinal form, the symptoms start in limb muscles, most frequently in the upper limbs.

Determining the progress rate of the disease is a significant prognostic and preventive factor. It is the key to planning the optimum moment to introduce an alternative feeding method (PEG) and when to apply mechanical ventilation to avoid complications in the form of choking and chemical pneumonia or the consequences of respiratory failure [6]. Disease monitoring is also necessary in clinical trials and medical experiments concerning new therapies, affecting the course of the disease [7]. Main known tool to monitor ALS is Amyotrophic Lateral Sclerosis Functional Rating Scale-Revised (ALSFRS-R) [8, 9].

This work is important for two main reasons. First of all, we made the first on such a scale validation of the reliability of the ALSFRS-R scale in Poland. Secondly, we have checked it in the situation of dynamic change in health, which is during experimental therapy with stem cells. As it turned out, the scale is a reliable tool in Polish conditions, but it seems that it might require some changes, which will be discussed later. ALSFRS-R is also a satisfactory tool to monitor the therapy used in patients with ALS but requires special attention at several measurement points.

## Materials and methods

The health status of 60 patients was assessed with ALSFRS-R tool between 4 and 7 times, which gives a total of 327 observations. The data was complemented by the results of the neurologopedic test which was conducted 3 times. Patients’ inclusion criteria were based on El Escorial World Federation of Neurology standards [10].

The data have been obtained during the medical experiment conducted by Instytut Terapii Komórkowych S.A. (Cell Therapies Institute, ITK) in Olsztyn, Poland, in cooperation with the Department of Neurology and Neurosurgery, School of Medicine, Collegium Medicum—University of Warmia and Mazury in Olsztyn, Poland, and the University Clinical Hospital in Olsztyn, Poland [11]. The ITK study was controlled by the Bioethical Committee of School of Medicine, Collegium Medicum—University of Warmia and Mazury in Olsztyn, Poland. (Ethical approval consent was given by the resolution no. 36/2014 of June 2014 and no. 8/2016 of February 2016.)

Socio-demographic features of the respondents and their results on each indicator of the ALSFRS-R are presented in Table 1 below (two versions of the question concerning preparation of food, corresponding to the distinction between patients with gastrostomy and without gastrostomy, were combined into one variable used for statistical analyses).

**Table 1:** Patients’ socio-demographic data and ALSFRS-R scores

Patient’s age	57.5 ± 8.6
Sex (female/male)	(23/35)
Speech	3.4 ± 1.1
Salivation	3.5 ± 1.2
Swallowing	3.6 ± 0.7
Handwriting	3.0 ± 1.3
Eating	3.1 ± 1.3
Dressing	2.8 ± 1.4
Turning in bed and adjusting sheets	3.4 ± 0.7
Walking	2.8 ± 1.1
Climbing stairs	2.6 ± 1.5
Dyspnoea	3.7 ± 0.8
Orthopnoea	3.7 ± 0.9
Respiratory insufficiency	3.9 ± 0.4

ALSFRS-R questionnaire was translated using the cross-translation method, by two independent translators and in consultation with a neurologist. The final tool was used, first of all, in the form of a survey completed by the researcher. The main method of conducting the interview was individually by telephone. The exception were Polish-language patients located abroad, who completed the survey on their own via the Internet. In a few cases, the survey was also sent via e-mail to Polish-language patients when contact by phone was not possible. Apart from ALSFRS-R scale, researcher also noted additional qualitative information about patients’ health and social problems.

ALSFRS-R tool evaluates the function of speech, swallowing, self-service abilities and patient mobility. It consists of 12 questions (cutting food is additionally differentiated for patients with and without gastrostomy), for which 5 answers are possible. The highest answer, representing the absence of a deficit in a given area, corresponds to 4 points, while the lowest answer, signifying the highest deficit in the examined area, corresponds to 0 points. Therefore, the patient’s health can be assessed on a scale ranging from 48 to 0 points [12].

According to literature, we have used four-factor structure to analyse areas examined by the ALSFRS-R [8, 13–17]: speech and swallowing (speech, salivation, swallowing), fine motor skills (handwriting, eating, dressing and hygiene), gross motor skills (turning in bed and adjusting sheets, walking, climbing stairs) and respiratory functions (dyspnoea, orthopnoea, respiratory insufficiency).

## Results

For the statistical evaluation of the reliability of ALSFRS-R questionnaire, two methods were applied: exploratory factor analysis and Cronbach’s alpha. In the first step, it was evaluated whether the ALSFRS-R indicators measure the corresponding life functions and skills in the same way and whether each indicator is validly related to only one life function or skill. For this purpose, the factor analysis was carried out using R software, with the default “minres” method for factor extraction and the “varimax” rotation. In the second step, for each life function/skill the Cronbach’s alpha statistic was calculated. This statistic informs us whether the empirical items derived from the ALSFRS-R questionnaire can be considered as valid and reliable instruments for assessing patients’ health status in each dimension.

**Fig. 1:**
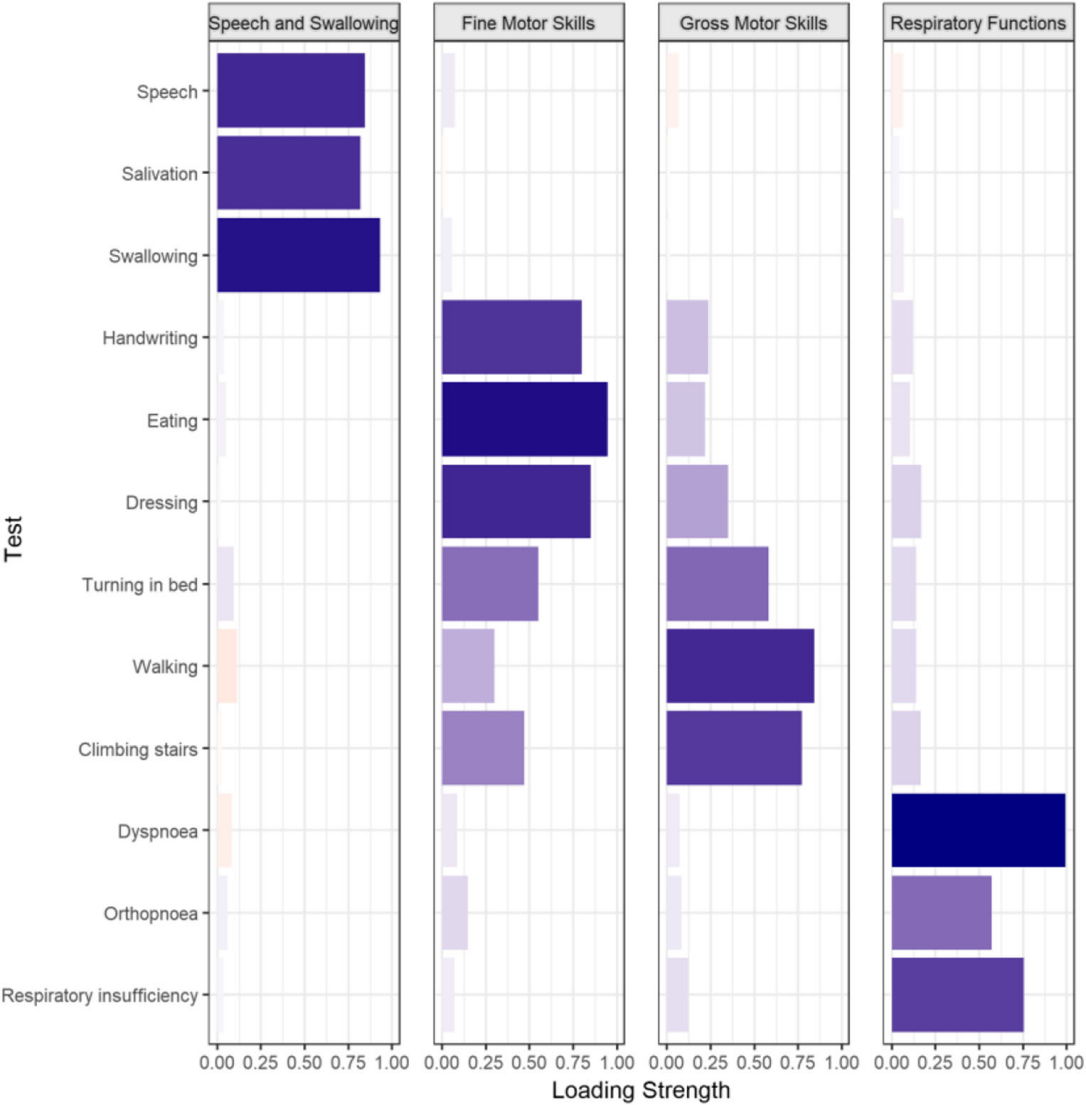
Exploratory factor analysis: factor loadings. Note: Figures in the parentheses inform on each indicator’s portion of variance explained by the unobserved factor

Figure 1 presents results from the performed factor analysis. It provides information on the general pattern of relationship between each empirical variable and the unobservable life functions and skills. The lengths of the bars (the vertical axis) represent factor loadings which inform us about the strength of the link between each variable and the corresponding factor. From the figure, we generally get the impression that variables such as “Speech”, “Salivation” and “Swallowing” load solely on the speech and salivation factor, while “Dyspnoea”, “Orthopnoea” and “Respiratory Insufficiency” load almost entirely on the respiratory functions factor. This intuition is confirmed when inspecting the data concerning the proportion of variance of each empirical variable that is explained by its respective unobserved factor. These are derived as the square root of factor loadings from the vertical axis. The speech and swallowing factor as well as the respirator function factor explain significant proportions of variances of their respective indicators.

The presented picture points to three potential sources of problems. First, in the case of the “Orthopnoea” variable, only 34% of its observed variance is explained by the respiratory life function factor. The remaining part of this variable variation remains unexplained by the proposed model. Second, the obtained results indicate that the fine motor factor contributes to explaining the variances of indicators ascribed to gross motor factor—i.e. it accounts for 31% of variance of patients’ scores on “Turning in bed and adjusting sheets” and 22% of variance of patients’ scores on “Climbing stairs”. Thirdly, the gross motor factor accounts for 12% of the observed variance of variable related to dressing, which, by default, is considered as belonging to the fine motor factor.

Although we observe some discrepancies in the way the common factors are related to the ALSFRS-R indicators, the general picture that emerges from the performed factor analysis supports the conclusion that the majority of empirical indicators are validly related to only one life function/skill. The relatively weakest fit is demonstrated by the “Orthopnoea” variable for the respiratory functions factor and “Turning in bed and adjusting sheets” variable for the gross motor skills factor. The calculation of the Cronbach’s alpha statistic will give the answer whether the link between the empirical indicators and the unobserved factors is strong enough to consider the tested scale as a reliable instrument.

Cronbach’s alpha is the measure of “internal consistency” between variables intended to measure the same unobserved (latent) variable. The measurement of a specific phenomenon using empirical indicators can be considered reliable only when empirical indicators are correlated to a high enough degree. Therefore, the value of the Cronbach’s alpha statistic depends on the average value of correlation between empirical indicators. It also depends on the number of items used in the measurement: the higher the number of variables used to measure a specific phenomenon, the higher the reliability of the measurement (intuitively: the lower is the chance that the result of the measurement is due to chance). The results presented below in Table 2 provide detailed diagnostics of the reliability analysis.

**Table 2:** Reliability analysis using Cronbach’s alpha

		Cronbach’s alpha if item deleted	Cronbach’s alpha for subscales
Speech and swallowing	Speech	0.86	0.95
	Salivation	0.88	
	Swallowing	0.81	
Fine motor skills	Handwriting	0.94	0.97
	Eating	0.88	
	Dressing	0.91	
Gross motor skills	Turning in bed and adjusting sheets	0.90	0.94
	Walking	0.84	
	Climbing stairs	0.80	
Respiratory functions	Dyspnoea	0.61	0.90
	Orthopnoea	0.87	
	Respiratory insufficiency	0.74	

The data presented in Table 2 provide, for each life function and skill, the value of Cronbach’s alpha. These values indicate the level of internal consistency between empirical indicators measuring specific unobserved factors. The estimated values of Cronbach’s alpha range from 0.82 (for the respiratory functions dimension) to 0.94 (for the fine motor skills dimension). Thus, they can be considered very high, safely exceeding the critical values of 0.7—the level regarded as minimum to draw a conclusion on scale reliability. The square roots of the alpha coefficients indicate the estimated level of the correlation between the unobserved factors and the indices created by averaging respective empirical indicators. Thus, the correlation between the speech and swallowing latent variable and the index created by averaging “Speech”, “Salivation” and “Swallowing” variables amounts 0.95. Respective correlations for other latent variables amount to 0.97 for fine motor skills, 0.94 for gross motor skills and 0.90 for respiratory functions.

Table 2 presents one more interesting information in terms of the results of exploratory factor analysis. It provides the values that the alpha statistics would reach if specific items would be removed from the scale (“Cronbach’s alpha if item deleted”). For each and every empirical item, the value of the statistic would decrease if the item would be removed from the scale. Thus, although the results of the factor analysis suggested that the “Turning in bed” variable was related both to gross motor latent variable and to the fine motor latent variable, the value of the Cronbach’s statistic if this item was deleted from the scale assures us that this item is rightfully in the scale. The same concerns the “Orthopnoea” variable. Deleting this item from the scale does not improve the internal consistency between the empirical variables forming the index.

Overall, the results of the analysis are reassuring in terms of reliability of the ALSFRS-R as a tool in assessing the patients’ health conditions. First, the estimated averaged values of Cronbach’s alpha are high enough to claim that the empirical indicators can be used to assess the patients’ performance in terms of speech and salivation, fine motor and gross motor skills as well as respiratory functions. Second, none of the estimated Cronbach’s alpha statistics could be higher by omitting one of the employed empirical indicators. Last but not least, the square roots of the estimated Cronbach’s alpha statistics inform on the high level of correlation between unobserved skills and life functions and indices formed by averaging empirical indicators.

### Neurologopedic measurement

Most of ALS patients in advanced stage are not able to communicate on everyday basis using natural speech [18]. The most significant effect of early damage to central motor neurons is the occurrence of motor disorders. They are a characteristic causative mechanism of speech disorders—dysarthria, and as the disease progresses, leading to anarthria and swallowing disorders, occurring regardless of food type or consistence [19–22]. For this reason, neurologopedic measurement was carried out in parallel to the study using the ALSFRS-R. In this study, this measurement was used as to validate the questions of the ALSFRS-R concerning speech and swallowing.

A neurologopedic test was carried out using a Frenchay Dysarthria Assessment (FDA), commonly applied for the clinical evaluation of speech disorders [23–25]. The intensity of dysarthria is described by the overall result obtained from evaluating individual speech levels under examination: articulation, articulatory motor functions, reflexes, respiration, phonation and prosody, all evaluated according to the 5-degree scale. Individual degrees of performance were described as follows: 4—normal, 3—quite good, 2—satisfactory, 1—poor, 0—unsatisfactory.

For the purpose of validating the results of the ALSFRS-R in this study, the value evaluating the condition of dysarthria is the result obtained by the patient for speech intelligibility—spontaneous speech. This function reflects to the fullest extent the functional condition of the articulatory competence of the examined person, also evaluated in the ALSFRS-R in question 1 (“Speech”). On the other hand, the value assessing swallowing is the result obtained in the FDA test with reference to the function of reflexes—swallowing, where a maximum score of 4 is given for swallowing without problems, and the lowest, 0—when the patient is not able to swallow. Additionally, the result obtained in the EAT-10 Questionnaire (from 0—swallowing with no problem to 4—severe problem) was used in order to increase the objectivity of the swallowing performance evaluation. These data in the aggregated form were compared to question 3 in ALSFRS-R (“Swallowing”).

As long as the subjective measurement of the life functions of patient is reliable, we should observe a high level of correlation between questions of the ALSFRS-R questionnaire and the results of the neurologopedic measurement. The existence of a high correlation between the evaluation of speech and swallowing function obtained from neurologopedic tests is indicated by the high coefficients of correlation presented in the Table 3. The obtained Spearman’s correlation coefficients are very high—for speech, the correlation coefficient amounts to 0.86, and for swallowing, it amounts to 0.90, at a level of statistical significance in both cases lower than 0.001. This means that the subjective evaluation of life functions (made by patient) corresponds to the results of neurologopedic measurements performed at the same time. Consequently, the Polish version of the ALSFRS-R questionnaire can be considered a reliable tool to evaluate the health condition of patients suffering from ALS.

**Table 3:** Correlations of ALSFRS-R questionnaire questions with the results of the neurologopedic measurement: Spearman’s correlation coefficients (and their statistical significance)

	Speech	Swallowing
Speech	0.86 (0.00)	
Swallowing		0.90 (0.00)

## Summary and discussion of the results

The ALSFRS-R scale is an important tool in examining the progress of amyotrophic lateral sclerosis [26] as well as monitoring the effects of therapy [27–32]. Our study confirms that the scale can be considered a reliable tool in the process of examining the state of the patients’ life functions in Poland. The values of the Cronbach’s alpha statistics estimated for individual life functions/skills of patients with a significant “reserve” exceed the level of 0.7—generally considered as the minimum to draw conclusions on the reliability of the measurement tool [33]. In addition, comparing the results from the scale with neurologopedic measurements further confirms the high reliability of the measurement. However, challenges related to the structure of individual questions and cultural differences, whose change could further improve the already high reliability of the ALSFRS-R scale, require further analyses.

Primarily, the interviewed person and the method applied are important for the examination performed. For the medical experiment, most interviews were carried out directly with a patient by telephone which seems to be satisfactory, as other studies have shown, even if assessment was made with a caregiver [34, 35], in contrast to e-mail, when it is difficult to ask the patients about their functions. An example is holding a pen—a variable measuring the strength of hand muscles. A patient who stops writing usually resigns from holding the pen in their hand, considering themselves in the context of the “Handwriting” to be helpless. However in this situation researcher can check whether the patient is actually able to hold the pen. It can have a great effect on evaluation of this function, changing the score from helplessness (0 points) to at least a vestigial existence of a given function (1 point). Attention should also be paid to certain cultural aspects, such as the preparation of food in our study group. Many patients, particularly men, indicated that they did not prepare meals on their own. When asked about it, they claimed that they could do it, but usually, the meals were prepared by others, e.g. their wives.

Moreover, the reliability of the retrospective measurement shall be discussed here. During the first interview with the patient, researchers carried out a retrospective analysis to reconstruct the course of illness at three additional time points: 2, 4 and 6 months earlier. Statistical analyses do not indicate that the retrospective measurement could have a negative effect on the reliability of the measurement. Therefore, such an additional measurement is the right way to construct a broader image of the history of the disease.

The obtained results point also three potential difficulties posed by the ALSFRS-R for explicit evaluation of certain functions. (1) For the variable concerning climbing stairs, classified into the gross motor skills dimension, part of its variance (22%) is explained by the latent variable fine motor skills. This situation can prove the fact that some skills belonging to the fine motor skill dimension inevitably participate in patients’ performance of activities belonging to the gross motor skills dimension. Climbing stairs requires not only the performance of the gross motor skills function but also a certain capability of the hand to hold onto the hand rail, which was frequently signalled by patients in an interview with the researcher. (2) The second potentially problematic variable is “Turning in bed and adjusting sheets”. The variation of this variable is explained equally by fine motor skills factor and gross motor skills factor. In other words, this factor could be equally considered the measure of patients’ abilities in the scope of fine motor skills factor for the manual activity of adjusting sheets and gross motor skills factor to turning the entire body. Perhaps the structure of the question itself determines such results. The question might seem to be of double nature—it asks the patients to make an evaluation of the degree of difficulties in performing two different activities. (3) There are also some minor issues related to patients’ difficulties with breathing when lying flat on their back (“Orthopnoea”). The analysis of reliability indicates that this is the variable that is the least adjusted to the latent variable which it is intended to measure. However, the value of the Cronbach’s alpha statistics estimated for the respiratory functions dimension (0.82) taking into account this variable is high enough to consider the measurement as reliable, at least with regard to the conventional threshold value of Cronbach’s alpha of 0.7.

The difficulties described in relation to three questions described above indicate the possibilities of improving them in order to obtain even more uniform factor structure. The factor analysis indicated that those questions are either related to more than one unobserved phenomenon or measure the latent variable in a manner standing out from other variables in a given dimension. It should be noted that the problem of multidimensionality in the ALSFRS-R was described before. Franchignoni et al. showed that lack of unidimensionality of the scale indicates that it should be partially revised [36]. However, those difficulties are not reflected in the estimated Cronbach’s alpha statistics. The values of these statistics are high enough to consider the measurement performed as being reliable. Any attempts to change the questionnaire should therefore concern improvement of the listed questions rather than omitting them at the stage of preparing indicators.

## Electronic supplementary material

ESM 1 (PDF 65.2 kb)

## Data Availability

Data material is available on request, which should be directed to the corresponding author. Validated ALSFRS-R scale in Polish is available to download online or directly from corresponding author upon request (Supplementary material 1)

## References

[1] Gordon PH (2011). Amyotrophic lateral sclerosis: pathophysiology, diagnosis and management. CNS Drugs.

[2] Rosenbohm A (2018). Phenotypic differences of amyotrophic lateral sclerosis (ALS) in China and Germany. J Neurol.

[3] Park J, Do Y, Park J (2019). Under-recognized primary spontaneous pneumothorax in ALS: a multicenter retrospective study. Neurol Sci.

[4] Armon C, Gorelick PB AM (1994). Motor neuron disease. Handbook of neuroepidemiology.

[5] Clarke C, Howard R, Rossor M, Shorvon S (2016) Neurology: a Queen Square textbook. Wiley

[6] Russ KB, Phillips MC, Wilcox CM, Peter S (2015). Percutaneous endoscopic gastrostomy in amyotrophic lateral sclerosis. Am J Med Sci.

[7] Rutkove SB (2015). Clinical measures of disease progression in amyotrophic lateral sclerosis. Neurotherapeutics.

[8] Cedarbaum JM, Stambler N, Malta E, Fuller C, Hilt D, Thurmond B, Nakanishi A (1999). The ALSFRS-R: a revised ALS functional rating scale that incorporates assessments of respiratory function. BDNF ALS Study Group (Phase III). J Neurol Sci.

[9] Park J, Kim S, Park D (2020). Effect of edaravone therapy in Korean amyotrophic lateral sclerosis (ALS) patients. Neurol Sci.

[10] Brooks BR, Miller RG, Swash M, Munsat TL, World Federation of Neurology Research Group on Motor Neuron D (2000). El Escorial revisited: revised criteria for the diagnosis of amyotrophic lateral sclerosis. Amyotroph Lateral Scler Other Mot Neuron Disord.

[11] Barczewska M (2019). Safety of intrathecal injection of Wharton’s jelly-derived mesenchymal stem cells in amyotrophic lateral sclerosis therapy. Neural Regen Res.

[12] Appel V, Stewart SS, Smith G, Appel SH (1987). A rating scale for amyotrophic lateral sclerosis: description and preliminary experience. Ann Neurol.

[13] Bakker LA, Schröder CD, van Es MA, Westers P, Visser-Meily JMA, van den Berg LH (2017). Assessment of the factorial validity and reliability of the ALSFRS-R: a revision of its measurement model. J Neurol.

[14] Guedes K, Pereira C, Pavan K, Valerio BCO (2010). Cross-cultural adaptation and validation of Als functional rating scale-revised in Portuguese language. Arq Neuropsiquiatr.

[15] Abdulla S, Vielhaber S, Körner S, Machts J, Heinze HJ, Dengler R, Petri S (2013). Validation of the German version of the extended ALS functional rating scale as a patient-reported outcome measure. J Neurol.

[16] Bacci ED, Staniewska D, Coyne KS, Boyer S, White LA, Zach N, Cedarbaum JM, The Pooled Resource Open-Access ALS Clinical Trials Consortium (2016). Item response theory analysis of the Amyotrophic Lateral Sclerosis Functional Rating Scale-Revised in the Pooled Resource Open-Access ALS Clinical Trials database. Amyotroph Lateral Scler Frontotemporal Degener.

[17] Wicks P, Massagli MP, Wolf C, Heywood J (2009). Measuring function in advanced ALS: validation of ALSFRS-EX extension items. Eur J Neurol.

[18] Makkonen T, Ruottinen H, Puhto R, Helminen M, Palmio J (2018). Speech deterioration in amyotrophic lateral sclerosis (ALS) after manifestation of bulbar symptoms. Int J Lang Commun Disord.

[19] Beukelman D, Fager S, Nordness A (2011). Communication support for people with ALS. Neurol Res Int.

[20] Chełstowska B, Kuźma-Kozakiewicz M (2020). Biochemical parameters in determination of nutritional status in amyotrophic lateral sclerosis. Neurol Sci.

[21] Panebianco M, Marchese-Ragona R, Masiero S, Restivo DA (2020) Dysphagia in neurological diseases: a literature review. Neurol Sci. 10.1007/s10072-020-04495-2 10.1007/s10072-020-04495-2 PMC7567719 32506360

[22] Chio A (2002). Early symptom progression rate is related to ALS outcome: a prospective population-based study. Neurology.

[23] Enderby P (1980). Frenchay dysarthria assessment. Br J Disord Commun.

[24] Konstantopoulos K, Zamba-Papanicolaou E, Christodoulou K (2018). Quantification of dysarthrοphonia in a Cypriot family with autosomal recessive hereditary spastic paraplegia associated with a homozygous SPG11 mutation. Neurol Sci.

[25] Pawlukowska W (2019). Comparative assessment and monitoring of deterioration of articulatory organs using subjective and objective tools among patients with amyotrophic lateral sclerosis. BMC Neurol.

[26] Gordon PH, Miller RG, Moore DH (2004). ALSFRS-R. Amyotroph Lateral Scler Other Motor Neuron Disord.

[27] Mazzini L, Mareschi K, Ferrero I, Vassallo E, Oliveri G, Boccaletti R, Testa L, Livigni S, Fagioli F (2006). Autologous mesenchymal stem cells: clinical applications in amyotrophic lateral sclerosis. Neurol Res.

[28] Mazzini L, Fagioli F, Boccaletti R, Mareschi K, Oliveri G, Olivieri C, Pastore I, Marasso R, Madon E (2003). Stem cell therapy in amyotrophic lateral sclerosis: a methodological approach in humans. Amyotroph Lateral Scler Other Mot Neuron Disord.

[29] Oh KW, Moon C, Kim HY, Oh SI, Park J, Lee JH, Chang IY, Kim KS, Kim SH (2015). Phase I trial of repeated intrathecal autologous bone marrow-derived mesenchymal stromal cells in amyotrophic lateral sclerosis. Stem Cells Transl Med.

[30] Kim HY (2009). Efficacy and safety of autologous bone marrow-derived mesenchymal stem cell treatment in patients with amyotrophic lateral sclerosis. J Korean Neurol Assoc.

[31] Petrou P, Gothelf Y, Argov Z, Gotkine M, Levy YS, Kassis I, Vaknin-Dembinsky A, Ben-Hur T, Offen D, Abramsky O, Melamed E, Karussis D (2016). Safety and clinical effects of Mesenchymal stem cells secreting Neurotrophic factor transplantation in patients with amyotrophic lateral sclerosis: results of phase 1/2 and 2a clinical trials. JAMA Neurol.

[32] Siwek T (2018). Mesenchymal stem cell (MSC) transplantation in patients with amyotrophic lateral sclerosis (ALS): is there a ‘responder population’?. J Neurol Neurosci.

[33] Bujang MA, Omar ED, Baharum NA (2018). A review on sample size determination for Cronbach’s alpha test: a simple guide for researchers. Malays J Med Sci.

[34] Kaufmann P, Levy G, Montes J, Buchsbaum R, Barsdorf AI, Battista V, Arbing R, Gordon PH, Mitsumoto H, Levin B, Thompson JLP, Kaufmann P, Levy G, Montes J, Buchsbaum R, Barsdorf AI, Battista V, Arbing R, Gordon PH, Mitsumoto H, Levin B, Thompson JLP (2007). Excellent inter-rater, intra-rater, and telephone-administered reliability of the ALSFRS-R in a multicenter clinical trial. Amyotroph Lateral Scler.

[35] Mannino M, Cellura E, Grimaldi G, Volanti P, Piccoli F, La Bella V (2007). Telephone follow-up for patients with amyotrophic lateral sclerosis. Eur J Neurol.

[36] Franchignoni F, Mora G, Giordano A, Volanti P, Chiò A (2013). Evidence of multidimensionality in the ALSFRS-R scale: a critical appraisal on its measurement properties using Rasch analysis journal of neurology. Neurosurg Psychiatry.

