# Supplementary material for: Validation of the revised Amyotrophic Lateral Sclerosis Functional Rating Scale in Poland and its reliability in conditions of the medical experiment
Source: Neurol Sci. 2020 Jul 16;42(3):943–9. doi: 10.1007/s10072-020-04565-5 (PMC7870618; doi:10.1007/s10072-020-04565-5)
Supplement: Supplementary file 1 — (PDF 65.2 kb) [file 10072_2020_4565_MOESM1_ESM.pdf]

Skala oceny funkcjonalnej w stwardnieniu zanikowym bocznym, wersja po rewizji (ALSFRS-R)

Cytowanie: S. Maksymowicz, P. Kukołowicz, T. Siwek et. al. 2020

Adaptacja: S. Maksymowicz, P. Kukołowicz, T. Siwek, A. Rakowska, Validation of the revised Amyotrophic Lateral Sclerosis Functional Rating Scale in Poland and its reliabilty in conditions of the medical experiment, Neurological Sciences (2020), DOI: 10.1007/s10072-020-04565-5

1. Mowa

Mowa prawidłowa

Zauważalne zaburzenia mowy

Mowa zrozumiała po powtarzaniu

Mowa połączona z niewerbalnymi sposobami komunikowania się

Utrata zdolności zrozumiałej mowy

2. Ślinienie

Normalne wydzielanie śliny

Nieznaczny ale wyraźny nadmiar śliny w ustach, może występować nocny ślinotok

Umiarkowany nadmiar śliny, może występować minimalny ślinotok w ciągu dnia

Znaczny nadmiar śliny z umiarkowanym ślinotokiem

Znaczny nadmiar śliny, konieczność stałego używania chusteczek

3. Połykanie

Normalne nawyki żywieniowe

Wczesne problemy podczas jedzenia – zdarzające krztuszenie się

Zmiana konsystencji spożywanych posiłków

Potrzeba uzupełniającego dokarmiania przez sondę

Wyłącznie żywienie pozajelitowe lub dojelitowe (NPO – *nil per os*)

4. Pisanie ręczne

Normalne

Powolne lub niedbałe, ale wszystkie słowa są czytelne

Nie wszystkie słowa są czytelne

Zdolność do utrzymania długopisu, ale niezdolność do pisania

Niezdolność do utrzymania długopisu

5a. Przygotowywanie jedzenia

Normalne

Nieco powolne i niezdarne, ale bez pomocy

Zdolność do przygotowania posiłku, ale w niezdarny i powolny sposób oraz z pewną pomocą

Posiłek musi zostać przygotowany przez inną osobę, ale zachowana jest zdolność do powolnego spożycia posiłku

Konieczność karmienia przez inną osobę

5b. Przygotowanie jedzenia (pacjenci z gastrostomią)

Normalne

Niezdarne, ale możliwe do wykonania bez pomocy

Potrzebna jest pomoc, głównie przy zamknięciach i łącznikach

Posiłki prawie całkowicie przygotowywane przez opiekuna

Niezdolność do wykonania jakiejkolwiek czynności w tym zakresie

6. Ubieranie się i dbanie o higienę

Normalne funkcjonowanie

Całkowicie samodzielne ubieranie się i mycie, ale z wysiłkiem lub niedokładne

Okresowo potrzebna pomoc lub stosowanie metod zastępczych

Potrzebna pomoc przy ubieraniu się i myciu

Całkowita zależność od opiekuna

7. Przewracanie się w łóżku

Normalne

Nieco wolne i niezdarne, ale bez potrzeby pomocy

Zdolność do samodzielnego przewrócenia się w łóżku lub poprawienia pościeli, ale z dużą trudnością

Możliwość rozpoczęcia, ale niezdolność do samodzielnego przewrócenia się w łóżku lub poprawienia pościeli

Bezradność

8. Chodzenie

Normalne

Wczesne trudności w chodzeniu

Chodzenie przy pomocy

Brak możliwości przemieszczenia się przy zachowaniu innych ruchów

Brak celowego ruchu kończyn dolnych

9. Wchodzenie po schodach

Normalne

Powolne

Łagodna niestabilność lub zmęczenie podczas wchodzenia po schodach

Wchodzenie jedynie z pomocą

Niezdolność do wejścia po schodach

10. Duszność

Nie występuje

Pojawia się podczas chodzenia

Pojawia się podczas wykonywania jednej lub kilku z wymienionych czynności: jedzenie, kąpiel, ubieranie się (codzienne czynności życiowe)

Pojawia się w spoczynku, trudności w oddychaniu występują podczas siedzenia lub leżenia

Istotne trudności w oddychaniu, rozważanie stosowania wspomagania oddychania

11. Duszność w pozycji leżącej na plecach (ortopnoe)

Nie występuje

Pewna trudność ze snem w nocy z powodu duszności. Nie ma potrzeby rutynowego używania więcej niż dwóch poduszek

Aby móc spać, potrzebna jest dodatkowa poduszka (więcej niż dwie poduszki)

Możliwość spania jedynie na siedząco

Niemożność spania

12. Niewydolność oddechowa

Nie występuje

Okresowe używanie BiPAP

Stałe używanie BiPAP

Stałe używanie BiPAP w ciągu dnia i w ciągu nocy

Inwazyjna wentylacja mechaniczna przy użyciu intubacji lub tracheostomii
